# Big Data Analytics in Supply Chain Management: A Qualitative Study

**DOI:** 10.1155/2022/9573669

**Published:** 2022-09-16

**Authors:** Basim Aljabhan, Melese Abeyie

**Affiliations:** ^1^Ports and Maritime Transportation Department, Faculty of Maritime Studies, King Abdulaziz University, Jeddah 21589, Saudi Arabia; ^2^College of Natural and Computational Science, Department of Chemistry, Injibara University, Injibara, Ethiopia

## Abstract

This work explores the leading supply chain processes impacted by big data analytic techniques. Although these concepts are being extensively applied to supply chain management, the number of works that examine and classify the main processes in the current literature is still scarce. This article, therefore, provides a classification of the current literature on the use of big data analytics and provides insight from professionals in the field in relation to this topic. A well-established set of practical guidelines was used to design and carry out a systematic literature mapping. A total of 50 primary studies were analysed and classified, chosen from a sample of 5, 437 studies after careful filtering to answer six research questions. In addition, a survey was prepared and applied by professionals working in the area. In total, 25 professionals answered a questionnaire with eleven questions, ten of which seek to explore the importance of big data analytics for the areas of the supply chain addressed in this work, and one intends to list the three areas where BDA can be more shocking. More than 60% of the studies are directly linked to the area of chain management; most studies performed empirical studies but rarely classified or detailed methodological procedures; almost 50% bring models to optimize some process or forecasts for better decision-making; more than 50% of professionals working in the area believe that the processes where big data analytics can effectively contribute are related to inventory and stockout management. This study serves as a basis for further research and future work, as it reviews the literature, pointing out the main areas that are being addressed and making a relationship with understanding these areas in practice.

## 1. Introduction

Technological breakthroughs are transforming data generation and analysis. According to Bumblauskas et al. [1], big data have the potential to alter management and the entire business process. This is why the transformation concept is so crucial. Kaynak et al. [2] define big data as a corporate resource with 5 Vs: volume, speed, variety, verifiability, and value. According to Kaur et al. [3], “volume” refers to the amount of data or physical space necessary to store it, which is expanding at an exponential rate, putting strain on existing storage systems. According to the same authors' definition of speed, how quickly data are transferred affects both data intake and production. Aye et al. [4] define a variety of data as data that can be created from a range of platforms. This means there are no standards; each platform generates data based on its structure. As a result, semi-organized and fully organized data can be generated. Elgendy et al. [5] define veracity as an IBM 2012 word that refers to data unreliability and is usually linked with information based on people's emotions or any other type of information dependent on human judgment. According to the same authors, Oracle defined value in 2012 and linked it to the concept of essential data, indicating that high-value information can be recovered through extensive data analysis. According to Mathrani et al. [6], veracity and value constitute the rigor of big data analytics (BDA). They are significant because other qualities of big data processing, such as volume, speed, and variety, would be useless without data analysis. Advanced analysis techniques are utilized to extract relevant information from large amounts of data, allowing for better decision-making. According to Gunasekaran et al., a variety of technologies, including sensors, barcodes, RFIDs, and Internet of things, are being utilized in supply chain management (SCM) to integrate and coordinate all the links in the chain (2016). The BDA is altering supply networks, and its use in supply chain management (SCM) has been reported on various issues. Since empirical evidence suggests that BDA in SCM can reduce operating costs and improve chain process agility, there is a growing interest in discovering the optimum sites and processes to implement BDA.

Several publications and literature reviews [7–9] and Mohammad Alsaffar et al. [10] examine big data analytic applications in the supply chain, with the vast majority focusing on operational settings such as factory production lines, product development, and product assembly, among other things. Chong [7]; Majidian et al. [8]; Solanki et al. [9]; and Abdulsattar Abdullah Hamad et al. [11] all reviewed the literature on material flow in industrial operations, with Borgi et al. [12] and Sengan and Set al [13] focusing on transport and logistics. According to Husamaldin et al. [14], few research studies have evaluated the supply chain from the perspective of BDA techniques. As a result, this study will employ a comprehensive mapping analysis and a survey to identify the supply chain regions where BDA has the most significant influence and develop a panoramic view of the current literature.

## 2. Theoretical Foundations

This section covers supply chain management, big data analytics, systematic mapping, and survey studies. These concepts are presented in such a way that they appear to be related to the project's purpose.

**Figure 1:**
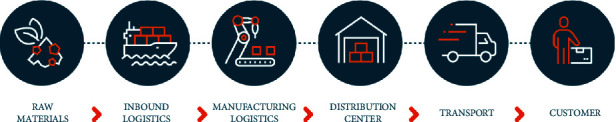
Supply chain.

Supply chain management is the management of the supply chain. According to Awwad [15], the term supply chain is used more frequently in the literature than the definition of supply chain management. According to Leveling [16], supply chains are made up of firms that move commodities. Multiple organizations collaborate as part of a supply chain to form an end-to-end procedure for manufacturing and delivering a product. This chain includes raw material and component producers, product makers, wholesalers, retailers, and transportation companies. Varela Rozados et al. [17] concur on the definition of a supply chain as the alignment of firms that deliver products or services to market.  Figure 1 displays various alternatives.

According to Felea et al. [18], SCM definitions fall into three categories: management philosophy, implementation, and management processes. Reference [19] suggests that supply chain management employs a systems approach, which views the chain as a coherent whole, rather than a collection of separate pieces. As a result, a new strategy known as supply chain management aims to coordinate the complete flow of commodities from maker to customer. It is a set of beliefs held by each organization in the supply chain that directly and indirectly impacts all other supply chain members and improves overall supply chain performance.

Albastroiu Nastase et al. [20] emphasize the importance of developing management practices that enable businesses to operate or behave in accordance with their management philosophy. As a result, several authors have written about supply chain management activities. They argue that businesses must widen their integrated behavior to encompass customers and suppliers to compete effectively today. Albastroiu Nastase defines supply chain management as the outer integration of integrated behaviors. In this context, supply chain management is defined as a set of actions that carry out the philosophy of chain management: chain management implementation. To meet the ever-changing expectations of the ultimate client, suppliers, operators, and manufacturers collaborate in a coordinated effort known as supply chain management.

In contrast to other authors, many authors, such as Lamba [21], emphasize the activities that comprise supply chain management. In other terms, a process is a collection of operations with a defined framework and measurable outcomes that collectively attempt to suit the objectives of a specific client or market. To put it another way, supply chain management, according to Saleem et al. [22] and Ahmed et al. [23], is a system for managing the movement of physical objects and associated data from the point of supply to the point of consumption to increase customer service and economic value.

*Big Data Analytics*. In contrast, Banik et al. [24] defined big data in 3 Vs in their definition of the concept. A new definition of big data was given by Lamba et al. [21]; it consists of five components: volume, speed, diversity, veracity, and value. Babiceano and Seker (2016) conclude from their analysis that the veracity and value aspects of big data, which represent elements related to the reliability and importance of information, are crucial for BDA because, without analysis, other big data processes would be of little value. Ramannavar [25] succinctly summarizes the BDA concept by referencing the use of sophisticated analysis techniques to extract useful information from vast amounts of data.

**Figure 2:**
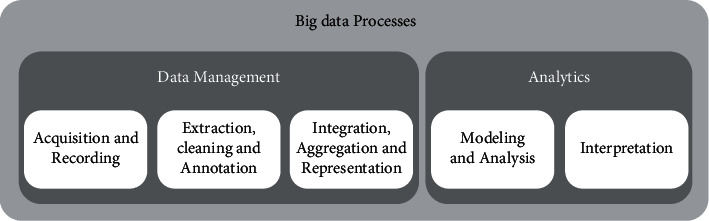
Big data processes [26].

According to Husamaldin [14], huge data are useless unless that can be interpreted. When big data are leveraged to guide business choices, its true value can be seen. To support evidence-based decision-making, organizations need to be able to transform vast volumes of speedy and diverse data into valuable information. Extraction, cleaning, and annotation; integration, aggregation, and representation; modeling and analysis; and interpretation are the five basic steps in the process of obtaining information from vast volumes of data. These five steps are divided into two main subcategories in Solanki [9]: analyses of data management and information storage. Data management refers to the processes and tools used to gather, store, prepare, and retrieve data for analysis. In contrast, “analytics” refers to the techniques used to study and gain understanding from important data. Big data analysis can be seen as a smaller phase in the larger process of extracting information from pertinent data, as seen in Figure 2.

Systematic Survey and Mapping Study (SSMS). According to Kapliński [26], SSMS aims to analyse a wider range of studies and rate the best representative studies on particular topics. In its study, SSMS primarily addresses issues connected to certain courses. Examples of these questions include those relating to research areas, empirical approaches, frequently utilized techniques, and the degree of automation of such techniques. On the other hand, surveys, according to Varela Rozados et al. [17], seek to understand the population from whom sampling was done. The company's largest developer population of 100 can be surveyed when 25 developers are questioned about a new procedure. Surveys are intended to produce generalizable findings. According to the authors, surveys may provide a huge number of elements to analyse. Still, this reduction in variables is necessary to estimate the greatest comprehension by the fewest variables because it makes the data collection and analysis process easier.

SSMS, according to Borgi et al. [12], offers a wider range of applications than other emergency medical services. The SSMS considers a large number of papers; however, only classification data are generated on these investigations. The classification and combining of research in SSMS are done using a preformatted classification technique. Researchers frequently elaborate on these categories, such as the type of technique, study location, publishing type, and research method employed, based on the information supplied in the papers. Habib et al. [19] contend that surveys can be used as a pre-study for a more in-depth investigation. Not providing an answer to the original research question can open up new opportunities for analysis. In contrast, surveys can be used to make statements about some populations, to explain how well people understand certain subjects, and even as a pre-study.

For a number of reasons, SCM while operating an SSMS is pertinent to this topic. To start, the method ensures that the literature review is impartial, exhaustive, and verified. Second, it intends to locate, evaluate, and compile all pertinent studies by conducting a complete study of the literature on supply chain and BDA. It offers a summary of a research topic and indicates whether or not there are any subtopics with sufficient research to conduct other types of analysis as the third component. This study also includes classification and analysis of current literature on a thematic level, which may be used to identify research gaps and suggest some lines of inquiry for future studies. The results of this study will be used to guide future research toward addressing the gaps that have been found.

To get a more complete picture of whether the processes being kinetically evaluated are in line with the beliefs and attitudes of the general population, the data collected through the SSMS will also be compared to the survey results.

## 3. Methodology

*Research Objectives and Questions*. The goals of this work are twofold: (1) to organize the existing research on applying big data analytics to the supply chain into useful categories and (2) to identify the chain processes where BDA has been applied most frequently. Based on this, for the systematic mapping study, six research questions (RQs) were formulated; in this way, the various particularities of the objectives can be carefully explored. According to Ramannavar [25], RQs in mapping studies should be generic to discover research trends over time and topics covered in the literature.  Table 1 summarizes the PQs as follows.

In addition, a survey was carried out taking into account the main areas of application of big data analytics in the supply chain to identify, based on the experiences of professionals in the area, which of these have the greatest impact on the chain. Lamba et al. [21] cite that a survey is a system to collect information from or about people to describe, compare, or explain their knowledge, attitudes, and behavior. Based on this, the questions shown in Table 2 were developed.

### 3.1. Mapping Study

This section aims to present how the review of the mapping study will be carried out. The first subsection, search strategy, deals with the search strategy used to obtain relevant studies on the topic. The second subsection, inclusion and exclusion criteria, describes the ways used to filter the studies. The third subsection, filtering the works, deals with how the works were filtered throughout the process to obtain the most relevant studies.

Search Strategy. As a means to accomplish the desired outcome, we shall specify the number of search engines. To ensure that search engines provide a more comprehensive result set, we will develop techniques based on a string structure that defines both the keywords and some of their synonyms.

Before searching for articles, it is essential to identify an effective set of keywords to capture the synthesis of existing literature related to our research topic. For this, the keywords are as follows:

1. Supply chain management/Supply Chain/Management/Manufacture/Sale/Inventory/Logistics/Requests/Transport
2. Big Data/Data/Data Volume/Data Speed/Data Variety/Data storage
3. Data Analysis/Data Mining/Descriptive Analysis/Predictive Analysis/Prescriptive Analysis/Machine Learning

From the definition of the groups mentioned above, the following string was created ((“Supply Chain” OR Manufacturing OR “Order Picking” OR Logistics) AND (“Big Data Analytics” OR “Predictive Analytics”)). Inclusion and Exclusion Criteria. This section aims to establish exclusion and inclusion criteria used to filter studies retrieved from selected electronic databases. The following list specifies the exclusion criteria (EC) defined. EC excluded studies that

- EC1: the purpose of this section is to define the criteria that were used to pick relevant studies from specific electronic databases. A list of the defined exclusion criteria (EC) is as follows.
- EC2: patent registration or early-stage projects where an overview and roadmap are presented are examples
- EC3: no keywords from the search string were present in the title, and the title's meaning runs counter to the inquiries posed in the aforementioned research questions
- EC4: no part of the study topics was addressed in the abstract
- EC5: appeared in duplicate
- EC6: did not address big data analytics or supply chain issues. On the other hand, inclusion criteria (IC) were used to add work to our sample.
- IC1: articles, final papers, master's and doctoral theses, or even dissertations focused on big data analytics or supply chain
- IC2: was published or disseminated
- IC3: studies published or available in scientific journals, conferences, pages of research groups, or educational institutions
- IC4: published until December 2021

*Method of Selection*. This section will cover how we narrowed down the available research to identify the most relevant papers. According to Mathrani et al. [6], a larger number of articles may not be preferable if a smaller number of articles more truly represent the desired topic's population. The following filtering procedure is presented to demonstrate the strategies used to identify representative studies using this methodology:

- Step 1: begin your search. The search string is sent to digital libraries to collect search results.
- Step 2: the first filter. Any disparities discovered in the search results are removed. The EC1 and EC2 will be used for this. Calls for conference papers, special issues of periodicals, patent specifications, and reports with no peer-reviewed material are all examples of inappropriately retrieved works.
- Step 3: sort by title. Exclusion criteria, EC3, are used to filter studies.
- Step 4: sort by abstract. Research is filtered with EC4 in mind. To eliminate papers whose content is unrelated to the key questions addressed in the research questions, Table 1 was used.
- Step 5: combine. All of the filtered studies from the previous phase are grouped in one location.
- Step 6: remove duplicates. A study is often found in two or more libraries. EC5 is then used to remove all duplicates, guaranteeing that each study is unique.
- Step 7: Filtering of research using EC6 in the entire text, omitting papers that are irrelevant to the topic at hand addressed in the research questions.
- Stage 8: exemplary works. The final selection of the most relevant studies is defined.

**Figure 3:**
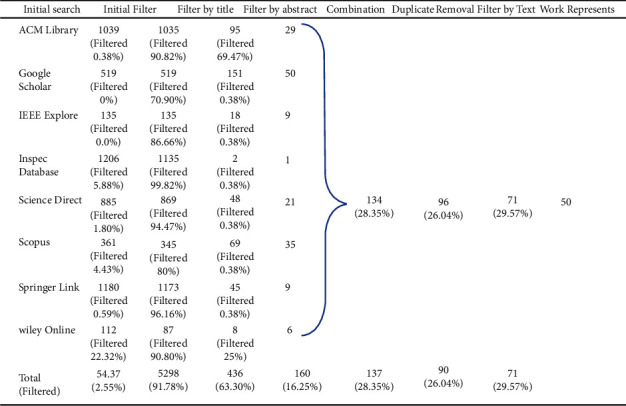
Results of the selection process.

*Filtering*. This section presents the execution of the filtering process defined in the previous section, where a total of eight steps were defined.  Figure 3 illustrates the result obtained in each step of the filtering process. The initial search for articles retrieved 5,437 articles, and then, the EC1 and EC2 were applied, with 2.55% (139 articles) discarded as impurities. Continuing the filtering process, the EC3 was applied to the other articles (5,298), with 91.77% (4,862 articles) being filtered through the title review. Then, EC4 was applied in a sample of 436 studies, where 63.30% (276 articles) were discarded after reviewing the abstracts. The remaining studies (160 articles) were gathered, and this combination generated a sample of 134 articles; that is, 16.25% of the articles were filtered at this stage. The next step was to eliminate duplicates, running EC5; this step reduced 28.35% (38 articles). Then, EC6 was applied, this stage of the process discarded 26.04% (25 articles). When examining 71 remaining works, some situations were observed in which the subjects were approached similarly: works produced based on previous articles. Thus, 29.57% (21 articles) were discarded. Finally, 50 articles were selected as the most representative of this study (Figure 3).

### 3.2. Survey

In addition to the mapping study, a survey was carried out, or in Portuguese, to identify, according to the participants' experiences and views, which areas and processes of the big data analytic supply chain have had the greatest impact. The following sections provide an overview of the preparation and execution of the survey.

*Selection of Participants*. The choice of participants was made through definitions of categories of specialists that make sense for the work, based on their practical and academic experiences, which is, therefore, the limiting factor for other professionals who are not inserted in this environment to participate in the study job. Participants must meet at least one of the following criteria:

- Be the owner/partner of a company operating in some area of the supply chain or of a technology company that provides products and/or services to companies operating in some area of the supply chain
- Be the manager of a company operating in some area of the supply chain or of a technology company that provides products and/or services to companies operating in some area of the supply chain
- Be a technology professional from a company operating in the supply chain area
- Be a strategic planning professional and know the supply chain
- Be a business consultant and have experience with supply chain companies
- Be a professor or researcher on big data analytics/supply chain.

*Preparation of the Questionnaire*. For the elaboration of the questionnaire applied to the study participants, the references acquired during the systematic mapping study were used, where the main points of impact for the supply chain were raised. From this, ten questions emerged to understand the relevance of big data analytics and also to compare the results between the opinions of professionals and the studies being developed in these areas. In addition to the questions directly related to the supply chain's areas of activity, another question was created for the participants to list, among the questioned areas, which, in their view, were the three most relevant when related to the use of big data analytics.

*Planning and Execution*. Given the construction of the questionnaire, it was necessary to find professionals who fit the study's participants' profiles. For this, it was decided to search for companies that act in this business segment and then make the questionnaire available to employees. A link was made available to answer the questionnaire through email and social networks to reach the participants.

## 4. Results

This section presents the results obtained through the mapping study and the survey in two subsections. In relation to the mapping study, each research question formulated in Table 1 will be discussed, and about the survey, the questions formulated in Table 2 will be addressed. For the results of the mapping study, the fifty most representative articles were taken into account. For the survey results, 25 responses obtained through the applied questionnaire were considered.

### 4.1. Systematic Mapping Study

RQ1: *Supply Chain Areas*. This question raises the areas of the supply chain where big data analytic concepts are used most frequently. The main results shown in Table 1 will be presented as follows. It can be seen that 78% or 39 of 50 articles evaluated focus on the areas of supply chain management and demand management. These results, concentrated in these two areas, are understandable, as they are areas where management and decision-making are constantly being exercised. BDA processes are applied to bring visibility and information to assist decision-making in these areas. In addition to this information, understanding the market's needs has been a very valuable differential; mastering this variable implies having effective control of production and avoiding large productions without demand, which consequently reduces large numbers of inventories and expenses with materials and unnecessary cousins. The opposite also applies in these cases, producing little for a lot of market demand. Representing 22% or 11 of 50 articles evaluated are the areas of manufacturing, transport/logistics, and storage/warehousing. These areas are directly linked to production and not management; they are areas where, in general, the focus of the application of BDA is aimed at optimization, whether by reducing time, cost, raw material, and other variables.

It is visible that BDA is being applied to management and control areas, as having information in decision-making brings a great competitive advantage; however, despite being little explored, the production areas of the chain have a lot to gain from applications of BDA, as many processes can be optimized, adding value to the product.

RQ2: *Levels of Big Data Analytics*. This research question raises at which levels BDA concepts are being applied most frequently. The main results shown in Table 2 will be presented as follows. Among the levels that were found, 66% or 37 of 50 articles evaluated represent the levels of predictive and descriptive analysis. These types of analysis are related to each other, while predictive analysis seeks to find future movements based on the data being reported by the most diverse platforms; the descriptive analysis seeks to find relationships and/or associations between historical data, and from these data, it predicts future movements. The levels of mixed and prescriptive BDA represent 34% or 17 of 50 articles evaluated; these two levels are somewhat generic, as the mixed level deals with the level of BDA that uses more than one technique to obtain the results, while the prescriptive deals with of tools and/or mechanisms used for analysis and presentation of information obtained by BDA techniques.

It is possible to see the trend of BDA levels for predictive and descriptive analysis, the advancement of technology, and the way we connect with it, which has been advancing over the years, and along with it, the amount of information that is generated by the most various means has been increasing exponentially, enabling processes to be created to analyse this lake of information and interpret and predict situations.

RQ3: *Big Data Analytic Models*. This research question raises the most frequently applied BDA models. Together, the optimization and prediction models represent 48% or 24 of 50 articles evaluated. The optimization model, in its essence, seeks through information to optimize some stage of the chain, whether by reducing time, cost, or processes. In contrast, the forecast model seeks to provide forecasts for better positioning, which is generally related to management and decision-making. Classification and simulation models represent 32% or 16 of 50 articles evaluated. The classification model lists the chain's main processes to focus on the most relevant processes.

In contrast, simulation processes focus on simulating future situations, usually in manufacturing processes, as they can predict and/or avoid possible problems. The other models represent 20% or 10 of 50 articles evaluated; they are visibility, mixed, and others. Despite being the models with less prominence, they are models that relate to each other by presenting techniques related to information management, with a focus on presenting the data. They bring a fundamental role that has already been seen in other sections; however, they are still little explored in the form of a BDA model.

Analysing the most used BDA models in the articles, it is possible to perceive a greater representation of the optimization and prediction models. However, the main focus of the BDA has been the management of information and aid in decision-making, and these models can indeed be used for this purpose, but it would be interesting to analyse why visibility reasons are being little used.

RQ4: *Big Data Analytic Techniques*. This research question raises the most frequently applied BDA techniques. Mixed techniques, visualization, and heuristic approach represent 68% or 34 of 50 articles evaluated. These techniques have a high representation because the mixed technique, as the name implies, uses two or more techniques to buy a BDA structure. The visualization technique is usually used to complement data mining techniques, one of the most used techniques in recent years; we can find this technique in most articles with mixed classification. On the other hand, the heuristic approach is widely used for optimization, and as this is one of the most used BDA models, it is understandable to use this technique on a large scale. The other techniques presented represent 32% or 16 of 50 articles. These are techniques that, despite their importance, are linked to very specific processes and therefore appear in smaller quantities; they are usually used in conjunction with other techniques.

It is possible to perceive a tendency to use techniques that we can call generic, as they can be applied at all stages of the chain, in addition to being techniques focused on providing information and optimizing processes. At the same time, specific techniques have been little used, despite providing a great differential.

RQ5: *Research Models*. This research question seeks to understand the research methods used by the selected studies. According to Mathrani et al. [6], studies can be categorized into six research methods: solution proposal, evaluation research, validation research, opinion article, experience article, and philosophical article [15]. Evaluation research models and solution research models represent 66% of 50 articles; these research models suggest that articles are being developed to evaluate existing processes in the chain and/or propose new solutions for them. The other models represent 34% of 50 articles evaluated. They suggest opinion models and/or validations of concepts and techniques applied in the studies.

Finally, it is possible to notice that works related to the subjects studied, for the most part, aim to evaluate concepts and techniques that are being used or provide our solutions for the processes. This behavior tends to happen due to the new technologies available on the market, as they open up many possibilities for improvement in processes.

**Figure 4:**
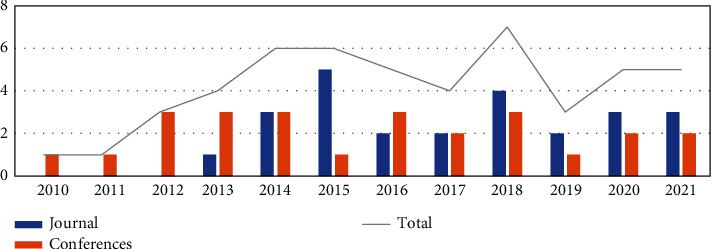
Research locations and year.

RQ6: *Research Location*. This research question investigates when and where the evaluated studies were published. The main objective is to examine the recent research trend of using big data analytics for supply chain processes. For this reason, the year of publication and the source for each selected study were collected.  Figure 4 provides a chronology for all the studies evaluated. The solid gray line in Figure 4 summarizes the number of evaluated studies published per year from 2010 to 2021. Although the period defined in the search strategy is until 2021, no studies were found before 2010. This may have occurred because big data had its first definition in 2001, but it was still very far from what we understand today; until one had a real understanding and usability of these emerging technologies, it may have taken a few years. Between 2011 and 2014, research on BDA for the supply chain experienced a considerable increase, but in 2016 with the great explosion of big data, research had the main peak.

It can be seen that in the years 2016 and 2021, the number of research related to the topic had considerable number. However, it will be necessary to follow the following years so that it can be affirmed that BDA has been widely used for supply chain processes since in 2021, where the polls suffered a slight drop.

### 4.2. Survey

Following the description of the data obtained, there is an analysis of the survey results so that it is possible to compare them with the results obtained in the mapping study. To focus on the analysis of the results, we will start with question number 11, where the results shown in Table 3 will be presented as follows. The main objective of this question is to list, among the areas of the supply chain, which participants consider BDA to have the greatest impact. Inventory management, stockout, and cost reduction together account for 67% or 50 of 75 responses, so questions regarding these three areas will be presented as follows.

Q1: *Inventory Management*. This question seeks to understand, in the view of experts, whether the supply chain inventory management process can be aided by the use of BDA. The main results shown in Table 4 will be presented as follows. Among the responses obtained, 84% or 21 of 25 responses fully agree that BDA can help this process, while 16% or 4 of 25 responses agree.

It can be seen that all participants agreed that this is one of the processes that can be most helped by the use of BDA; there were no neutral or negative responses. It remains a point of attention so that works and activities can be developed using this chain area.

Q9: *Disruption Management*. This question seeks to understand, in the view of experts, whether the supply chain disruption management and control process can be aided by the use of BDA. The main results shown in Table 5 will be presented as follows.

## 5. Discussion and Challenges

This study seeks not only to provide, through the mapping study, useful information to researchers and professionals interested in understanding which areas of the supply chain are most impacted by BDA but also to bring a perception of how these processes are being seen and used in practice, to create a relationship and address new challenges.

*Perception of Academic Outcomes vs. Professionals.* As can be seen through the interpretation of Tables 1 and 3, most academic results are focused on presenting BDA resources aimed at chain management, that is, methods that assist in decision-making in the most diverse processes; on the other hand, according to the sample carried out with professionals in the area, BDA has been used and/or in the perception of professionals, it can better help the areas of stock and rupture. This lack of connection between what is being researched and what is being applied in practice opens many doors for new investigations, whether focusing research and efforts in areas where the concepts are already being used, or applying these concepts in management processes. Applying, executing, and monitoring some of the techniques in practice should bring interesting results to be analysed and disseminated.

*Architecture for Real-Time BDA*. In the course of this work, a strong trend in the use of predictive techniques and machine learning can be analysed, where the strongest characteristics are the use of historical data creating relationships; however, there is a deficiency in techniques for using BDA in time. This tends to be a prosperous path for the future, as concepts of smart cities and other advances in the most diverse devices make information increasingly complete and more accessible, making everything very dynamic. More and more information is obtained in less time, and it is necessary that analysis techniques can keep up with this pace.

## 6. Conclusions

The study's purpose was to categorize the available literature and then list the primary supply chain areas where big data analytic approaches are used. The notion of conducting a survey and generating a sample for comparison was developed to establish a relationship between what is being researched and examined in the academic community.

Using the filtering approach used for the mapping study, a total of 50 articles were selected for analysis. These publications were then categorized based on the research questions. These research questions enabled it to deduce the course of the investigation. More than 70% of BDA concepts were discovered to be employed in managing some process in the supply chain, with the vast majority of that time spent controlling that process and the remainder focusing on demand management. A survey of 25 professionals, however, reveals a very different picture. BDA is particularly valuable to professionals in these areas.

First, the current literature was studied and classified; second, the supply chain areas most affected by BDA were identified; and third, the strategies used to execute BDA were provided. To put it another way, it is commonly acknowledged that these contributions have a substantial impact because they direct study in these areas and bring a sample of the population's understanding to the table.

## Figures and Tables

**Table 1:** SSMS research questions.

Research questions	Motivation	Variable
RQ1: In which areas of supply chain management is big data analytics being applied?	Discover which areas of supply chain management big data analytic approaches are being used extensively	Supply chain areas
RQ2: At what level of big data analytic analysis is used in these areas of chain management supplies?	List the main levels of *big data analytics* used for the supply chain management	Big *data levels*
RQ3: What types of big data analytic models are used in supply chain management?	Classify the *big data analytic models* used for the supply chain	*Big data models*
RQ4: What big data analytic techniques are used to develop these models?	List the main big *data analytic techniques used* in the models	*Big data techniques*
RQ5: What are the search methods used in the works?	List the most search methods used	Search methods
RQ6: Where were the studies published?	List the target vehicles used to disclose the results	Search location

**Table 2:** Survey questions.

Id	Question
Q1	Considering supply chain processes, do you consider important management from big data analytic techniques?
Q2	Considering supply chain processes, do you agree that big data analytics can assist in managing stock in transit?
Q3	Considering supply chain processes, do you agree that big data analytics can assist in vehicle routing (logistics)?
Q4	Considering supply chain processes, do you agree that big data analytics can assist in locating selection for installations?
Q5	Considering supply chain processes, do you agree that big data analytics can assist in selecting suppliers?
Q6	Considering supply chain processes, do you agree that big data analytics can assist in demand-driven storage?
Q7	Considering supply chain processes, do you agree that big data analytics can assist in real-time demand processes?
Q8	Do you agree that big data analytics can help reduce costs considering supply chain processes?
Q9	Considering supply chain processes, do you agree that big data analytics can help so that there is no lack of product in the gondola?
Q10	Considering supply chain processes, do you agree that big data analytics can assist in collecting orders? In the previous questions, relevant points for the supply chain were mentioned. Select 3 processes you think are the most important, that is, the processes in your perception
Q11	Have more impact along the chain

**Table 3:** Main supply chain processes.

Question 11	Answer number	Percentage
Inventory management	22	29
Lack of product in the gondola (rupture)	19	25
Cost reduction	9	12
Real-time demand processes	6	8
Demand-driven storage	5	7
In-transit inventory management	4	5
Vehicle routing (logistics)	4	5
Supplier selection	3	4
Order collection	2	3
Location for facilities	1	1
Total	75	

**Table 4:** Inventory management.

Question 1	Number of answers	Percentage
You fully agree	21	0.84
Agree	4	0.16
Neutral	0	0
Disagree	0	0
Totally disagrees	0	0
Total	25	

**Table 5:** Breakage management.

Question 9	No. of answers	Percentage
Totally agree	21	84
Agree	3	12
Neutral	1	4
Disagree	0	0
Totally disagree	0	0
**Total**	**25**	

Bold value mean number of Total Answers.

## Data Availability

The data underlying the results presented in the study are available within the manuscript.
